# Online Genetic Counseling as a Solution for Unmet Needs in Genetic Medicine: The First Survey in Japan

**DOI:** 10.31662/jmaj.2025-0157

**Published:** 2025-11-21

**Authors:** Haruka Murakami, Satomi Inoue, Kaoru Fujinami, Tatsuo Matsunaga, Kazuki Yamazawa

**Affiliations:** 1 Department of Medical Genetics, NHO Tokyo Medical Center, Tokyo, Japan

**Keywords:** online genetic counseling (OGC), telemedicine, genetic counseling, patient satisfaction, health care accessibility, clinical genetics

## Abstract

**Introduction:**

The demand for genetic counseling is increasing in Japan owing to rapid advancements in genetic medicine and increased utilization of genetic testing. However, access to genetic counseling remains limited, particularly in rural areas, owing to a shortage of certified professionals. Online genetic counseling (OGC), a form of telemedicine, offers a potential solution to address these disparities. Although OGC is widely practiced in Western countries, its implementation and systemic evaluation in Japan remain limited. To our knowledge, this study represents the first attempt in Japan to systematically assess the effectiveness, challenges, and user satisfaction of OGC compared with in-person genetic counseling (IPGC) in the context of the Japanese health care system.

**Methods:**

This cross-sectional, single-center study involved 49 participants (15 OGC, 34 IPGC) who received genetic counseling at the NHO Tokyo Medical Center between July 2020 and January 2025. Participants completed anonymous questionnaires assessing demographic characteristics, satisfaction with counseling, and perceived advantages and disadvantages. Statistical analyses included Mann-Whitney U tests, chi-square tests, and Fisher’s exact tests. Free-text responses were analyzed using conventional content analysis and word cloud visualization.

**Results:**

Overall satisfaction was high in both groups, with all participants selecting “Strongly agree” or “Agree” regarding satisfaction. However, the IPGC group scored significantly higher in counselor introduction, responsiveness, and overall satisfaction. OGC participants had significantly longer travel times and were more likely to be in their 20s-30s. Key advantages of OGC included convenience and accessibility, whereas disadvantages included concerns about privacy and technical issues.

**Conclusions:**

OGC has high potential to improve access to genetic services in Japan, particularly for individuals in remote areas. Despite high satisfaction, challenges such as communication limitations, privacy concerns, and lack of insurance coverage must be addressed. Policy reforms, improved infrastructure, and further large-scale studies are needed to support the widespread implementation of OGC in Japan.

## Introduction

Recent remarkable advances in genetic medicine have led to the development of various genetic tests, which are now widely used across the medical field to enable accurate diagnosis, appropriate treatment selection, and disease prevention ^(1)^. In Japan, the number of genetic tests conducted in clinical practice has increased dramatically, driving the growing importance and demand for genetic counseling ^(2), (3)^. However, as of February 2025, there are only 1,964 certified clinical geneticists in Japan (https://www.jbmg.jp/senmon/), and as of December 2024, the number of certified genetic counselors stands at just 428 (https://plaza.umin.ac.jp/~GC/About.html). These figures, when compared with the escalating demand and the situation in other high-income nations, highlight a significant shortage of specialists ^(2)^. This shortage of specialists further exacerbates the limited availability of genetic counseling services, which remain primarily concentrated in urban areas, creating significant disparities in access to genetic services nationwide ^(4)^. This disparity between the increasing need for genetic services and the limited, unevenly distributed capacity to deliver them constitutes a critical “unmet need” in Japanese genetic medicine.

Meanwhile, recent advancements in information and communication technology (ICT) have increasingly brought “telemedicine” into practical use in health care. In Japan, even before the coronavirus disease 2019 (COVID-19) pandemic, efforts were already underway to address issues such as medical staff shortages and regional disparities through ICT adoption and online medical consultations. In March 2018, the Ministry of Health, Labour and Welfare of Japan issued its first comprehensive guideline for the appropriate implementation of telemedicine services ^(5)^. This guideline has undergone several revisions in response to the accelerated adoption of telemedicine during the pandemic. Moreover, in December 2018, the first guideline for telemedicine in psychiatry was established ^(6)^. Psychiatry is particularly amendable to telemedicine adoption, given the field primarily relies on face-to-face interviews when practitioners and patients observe each other’s facial expressions. Similarly, genetic counseling, which emphasizes visual interaction and psychosocial support through interview-based sessions, also appears well-suited for telemedicine. Following this trend, online genetic counseling (OGC) has gained increasing international attention as a practical application of telemedicine in the field of medical genetics.

In Western countries, telemedicine has been gradually adopted since the early 2000s, and OGC has already become a routine practice ^(7), (8)^. Previous studies have suggested that OGC can effectively reduce inequities in access to genetic specialists in terms of time and cost ^(9), (10), (11), (12)^. In addition, patient satisfaction with OGC has been reported to be comparable to, or even higher than, that of in-person genetic counseling (IPGC) ^(7), (11), (13), (14), (15), (16)^. A 2024 survey conducted by the National Society of Genetic Counselors in the United States revealed that among 1,526 member respondents, 1,249 (81.8%) had experience providing OGC through audiovisual technology, indicating the widespread adoption of this practice ^(8)^.

However, to the best of our knowledge, OGC practice in Japan remained limited before the COVID-19 pandemic, with no major clinical studies or systematic case reports published. The pandemic promoted more medical genetics departments in Japan to adopt OGC, although most facilities are still in the trial-and-error phase ^(17)^. OGC has the potential to address the previously mentioned unmet needs in genetic medicine, including the increasing demand for genetic testing beyond current capacities, shortage of specialists, and their uneven geographical distribution, by leveraging telemedicine to connect patients with specialists remotely and reduce geographical and travel-related barriers. Meanwhile, challenges such as difficulties in nonverbal communication, billing and reimbursement issues, and technical connectivity problems have been reported from Western countries ^(9), (10), (18)^. Although OGC is increasingly practiced internationally, its implementation and systematic evaluation in Japan have lagged. Cultural factors, such as general hesitancy toward genetics-related topics and heightened privacy concerns ^(19), (20)^, combined with unique aspects of the health care system, highlight the need for research tailored to the Japanese clinical and social context. Although careful evaluation of the challenges, effectiveness, and safety of OGC is necessary for its widespread implementation, no comprehensive clinical studies on OGC have yet been conducted in Japan.

In this study, we conducted what we believe is the first clinical investigation of OGC in Japan, comparing OGC with IPGC through questionnaire surveys and statistical analysis. We hypothesized that OGC could contribute to reducing health care disparities in genetic medicine in Japan in the post-COVID era, as telemedicine enters a phase of widespread adoption. The aim of this study was to evaluate the effectiveness, challenges, and user satisfaction of OGC in clinical genetics settings to build evidence supporting its practical implementation.

## Materials and Methods

### Study design

This study used a single-center, cross-sectional, non-blinded design using anonymous questionnaire surveys. Owing to constraints in the environment and equipment availability, participant registration and group allocation were conducted in actual clinical genetic counseling settings to ensure an approximately equivalent distribution of numbers, conditions, and characteristics between the OGC and IPGC groups. Genetic counseling was provided by one certified clinical geneticist and one certified genetic counselor. After counseling sessions, questionnaires and research request forms were mailed to participants in the OGC group, whereas these forms were handed directly to those in the IPGC group. All responses were collected anonymously through postal mail. Data collection was conducted from July 15, 2020, to January 6, 2025. This study was approved by the institutional review board of the NHO Tokyo Medical Center (Approval Number: R22-115).

### Subjects

The study participants comprised clients and their relatives who received genetic counseling at the Department of Medical Genetics, NHO Tokyo Medical Center, Tokyo, Japan. The inclusion criteria were 1) age 20 years or older, and 2) provision of informed consent for study participation. Notably, the questionnaire introduction (Supplementary Documents 1 and 2) stated that results would be used solely to improve the quality of OGC. This introductory statement was intended to emphasize the quality improvement goal of the survey. All participants were fully informed and consented that their survey responses would be analyzed for research purposes under the approved study protocol.

### Environment for OGC

For communication devices, we used tablet devices such as iPads (Apple Inc., Cupertino, CA, USA) or personal computers. For communication software, we used Health Insurance Portability and Accountability Act-compliant (https://www.hhs.gov/hipaa/index.html) videoconferencing system Zoom (Zoom Communications, San José, CA, USA) or the online medical consultation system CURON (MICIN Inc., Tokyo, Japan), on the basis of the client’s communication environment. These systems were maintained separately from medical information systems such as electronic health record terminals. Security measures included regular antivirus software updates, operating system and software updates, and implementation of server certificates issued by trusted certificate authorities to ensure encrypted communications. For clients who had difficulty setting up their communication environments, we provided loaner tablet devices. Software and hardware logs and audit records were maintained throughout the process.

### Survey

The questionnaire included items regarding participant demographics, satisfaction with genetic counseling, communication devices and connection quality (OGC group only), and the advantages and disadvantages of both OGC and IPGC. The complete questionnaires used for both groups are provided in the Supplementary Documents 1 and 2. An outline of the questionnaire content is presented later.

#### Participant demographic characteristics

This section comprised six items: sex, age group, consultation content, relationship to the proband, number of genetic counseling sessions, and travel time to the hospital. All items followed a multiple-choice format, with ordinal scales for age group, counseling session frequency, and travel time to the hospital, and nominal scales for sex, consultation content, and relationship to the proband.

#### Satisfaction with genetic counseling

This section featured 11 questions about participants’ satisfaction with their genetic counseling experience, based on the Genetic Counseling Satisfaction Scale ^(21)^. Responses were measured on a five-point Likert scale.

#### Communication devices and connection quality (OGC group only)

This section included seven questions regarding the quality of communication devices and connectivity during OGC sessions. Responses were measured on a five-point Likert scale.

#### Advantages and disadvantages of OGC and IPGC

The survey concluded with open-ended questions about the advantages and disadvantages of both OGC and IPGC. In addition, a free-comment section was provided for participants to share their thoughts and suggestions regarding their genetic counseling experience.

### Data analysis

Survey responses were summarized using descriptive statistics. Frequencies and percentages were calculated for all items, and categories were maintained without consolidation during analysis to preserve response nuances. To compare the OGC and IPGC groups, Mann-Whitney U tests, chi-square tests, or Fisher’s exact tests were used. These analyses were performed using SPSS Statistics version 26.0 (IBM SPSS, Chicago, IL, USA). The dependent variable was the mode of genetic counseling (OGC or IPGC), whereas independent variables comprised participants’ demographic characteristics (sex, age group, consultation content, relationship to the proband, number of genetic counseling sessions, travel time) and the 11 questions regarding satisfaction with genetic counseling. For categorical data analysis, Fisher’s exact test was used instead of chi-square test when more than 20% of the contingency table cells had expected frequencies less than five. The statistical significance level was set at p < 0.05.

Free-text responses were analyzed using both conventional content analysis and text mining with word cloud visualization, in which more commonly appearing terms are displayed more prominently, providing a synopsis of the major themes ^(22)^. For the conventional content analysis, two authors (H.M. and S.I.) independently reviewed all free-text responses to generate initial codes and categories through an inductive process. These preliminary codes and categories were then discussed between the two authors, and any discrepancies were resolved by consensus or in consultation with a third author (K.Y., the corresponding author), leading to the development of a final coding framework. The same two authors subsequently applied this framework independently to code all responses. Regular discussions were held to ensure coding consistency. To ensure the validity and alignment with the overall study objectives, the entire research team reviewed the final categories and their corresponding responses. Moreover, word cloud analysis was conducted using NVivo 15 (QSR International, Melbourne, Australia) to visualize large amounts of text data and identify key themes and patterns.

## Results

### Participant demographic characteristics

A total of 49 participants responded to the survey: 15 in the OGC group and 34 in the IPGC group, with an overall response rate of 53.3%. The demographic characteristics of the respondents are listed in Table 1. To examine the differences in demographic characteristics between the OGC and IPGC groups, we performed Mann-Whitney U tests, chi-square tests, or Fisher’s exact tests as appropriate. The analysis revealed that travel time to the hospital was significantly longer in the OGC group (U = 101.5, p = 0.003). Specifically, although only 20.6% of the IPGC group reported travel times exceeding one hour, this proportion was markedly higher at 53.3% in the OGC group. Although no significant differences were observed for other characteristics, a notable difference was found in the age distribution: 53.3% of the OGC group were in their 20s or 30s, compared with only 26.4% in the IPGC group. The “Not specified” responses for some demographic items in Table 1, such as sex and consultation content, may reflect the heightened sensitivity to privacy among participants, potentially related to the urban location of our institution and the highly personal and sensitive nature of genetic information.

**Table 1.** Patient Demographic Characteristics.

		OGC (n=15)	IPGC (n=34)	p value
Sex, n (%)				0.244
	Male	5 (33.3)	5 (14.7)	
	Female	9 (60)	26 (76.5)	
	Not specified	1 (6.7)	3 (8.8)	
Age, n (%)				0.062
	20s	5 (33.3)	3 (8.8)	
	30s	3 (20)	6 (17.6)	
	40s	4 (26.7)	12 (35.3)	
	50s	2 (13.3)	10 (29.4)	
	60s	0 (0)	3 (8.8)	
	70s or older	1 (6.7)	0 (0)	
	Not specified	0 (0)	0 (0)	
Consultation content, n (%)				0.132
	Hereditary hearing loss	2 (13.3)	3 (8.8)	
	Hereditary retinal disease	5 (33.3)	2 (5.9)	
	Hereditary cancer	5 (33.3)	19 (55.9)	
	Congenital pediatric disorders	1 (6.7)	3 (8.8)	
	Others	1 (6.7)	3 (8.8)	
	Not specified	1 (6.7)	4 (11.8)	
Relationship to the proband, n (%)				0.295
	Self	10 (55.5)	28 (80.0)	
	Parent	2 (11.1)	3 (8.6)	
	Child	4 (22.2)	3 (8.6)	
	Sibling	0 (0)	0 (0)	
	Grandparent	1 (5.6)	1 (2.8)	
	Cousin	1 (5.6)	0 (0)	
	Others	0 (0)	0 (0)	
	Not specified	0 (0)	0 (0)	
Number of genetic counseling sessions, n (%)				0.459
	First	8 (53.3)	22 (64.7)	
	Second	4 (26.7)	9 (26.5)	
	Third	2 (13.3)	2 (5.9)	
	Fourth or more	0 (0)	0 (0)	
	Not specified	1 (6.7)	1 (2.9)	
Travel time to the hospital, n (%)				0.003*
	Under 30 minutes	2 (13.3)	14 (41.2)	
	30-60 minutes	3 (20)	13 (38.2)	
	1-2 hours	2 (13.3)	7 (20.6)	
	More than 2 hours	6 (40)	0 (0)	
	Not specified	2 (13.3)	0 (0)	

For the “Relationship to the proband” category, multiple responses were permitted. OGC, online genetic counseling; IPGC, in-person genetic counseling; *p < 0.05.

### Satisfaction with genetic counseling

**Figure 1.**
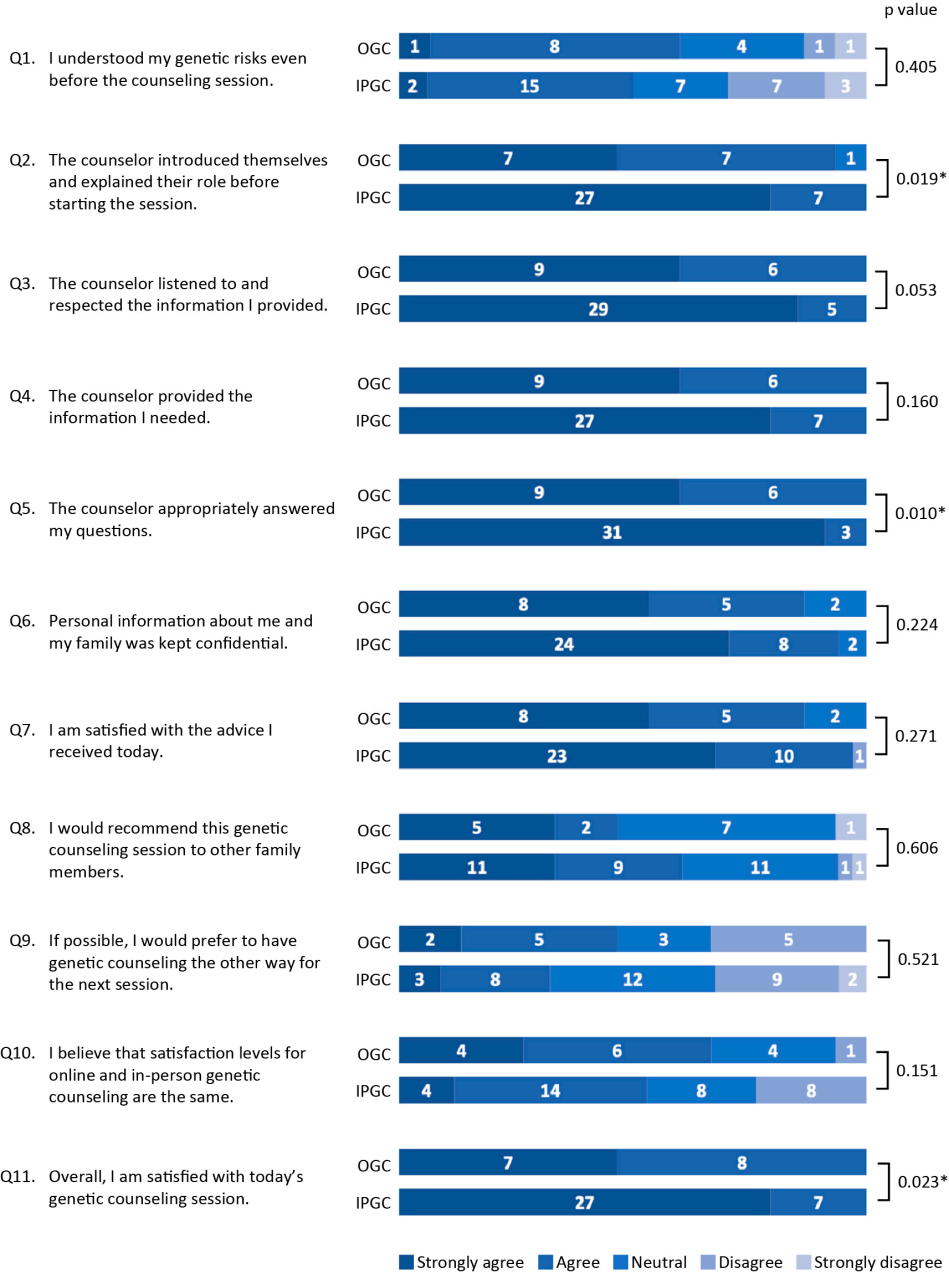
Satisfaction with genetic counseling in the OGC and IPGC groups. Participants rated their satisfaction with 11 modified questions from the Genetic Counseling Satisfaction Scale using a 5-point Likert scale. Numbers displayed represent responder counts. Statistical differences were analyzed using Mann-Whitney U test. IPGC: in-person genetic counseling; OGC: online genetic counseling; *p < 0.05.

Figure 1 presents the comparative satisfaction levels between the OGC and IPGC groups. Mann-Whitney U tests revealed significantly higher scores in the IPGC group for the following three questions:

Q2: The counselor introduced themselves and explained their role before starting the session (U = 168.0, p = 0.019)

Q5: The counselor appropriately answered my questions (U = 175.5, p = 0.010)

Q11: Overall, I am satisfied with today’s genetic counseling session (U = 171.5, p = 0.023)

Notably, for Q11, which assessed overall satisfaction with the genetic counseling session, all respondents in both the OGC and IPGC groups selected either “Strongly agree” or “Agree,” indicating that both groups generally found the sessions satisfactory. No significant differences were observed in the remaining questions.

### Communication devices and connection quality (OGC group only)

The responses from the OGC group regarding communication devices and connection quality are shown in Supplementary Table 1. More than 85% of participants selected “Strongly agree” or “Agree” when asked about the quality of audio and video during the session. Similarly, more than 85% of participants reported feeling comfortable asking questions during the OGC sessions. However, one participant expressed significant concerns about privacy and the risk of personal information leakage. None of the participants in this study used a loaned tablet. Therefore, many respondents likely selected “Not specified” for questions related to device delivery and usability (Q1, Q2, and Q7) because these questions were not applicable to their situation.

### Advantages and disadvantages of OGC and IPGC

**Figure 2.**
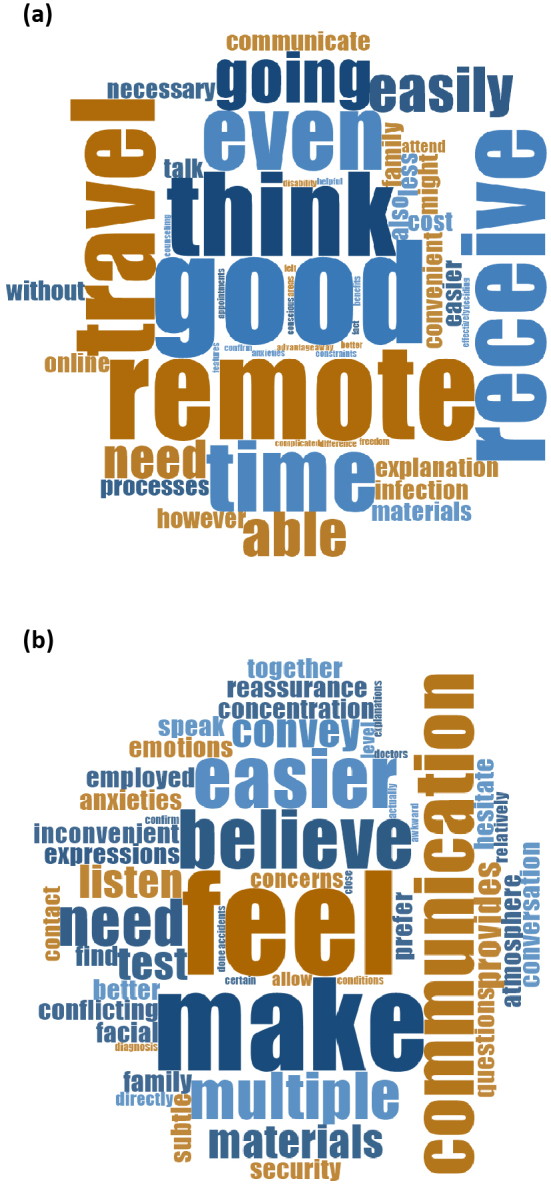
Visual representation of advantages of OGC (a) and IPGC (b) using word cloud. Word cloud for OGC highlighted terms related to practical advantages and convenience (a), whereas that for IPGC emphasized terms associated with communication and interpersonal aspects (b). The color-coding of terms serves no purpose other than visual distinction. IPGC: in-person genetic counseling; OGC: online genetic counseling.

Table 2 presents the categorization of advantages and disadvantages for OGC and IPGC. Frequently mentioned advantages of OGC included “Easy access even from remote areas,” “Saves time,” “Good communication possible,” and “No need to travel.” Other noted benefits were “Easy to receive genetic counseling,” “No risk of infection,” “Saves money,” and “Easier for family to join.” In contrast, disadvantages of OGC included “Difficulty ensuring privacy protection,” “Problems accessing online systems,” “Difficulty in communication,” and “Materials hard to see on device screens.” For IPGC, the most frequently mentioned advantage was “Emotions, facial expressions, and atmosphere are easily conveyed.” Other benefits related to communication were also included, such as “Smooth communication,” “Easy to ask questions,” and “Easier to speak honestly.” Disadvantages of IPGC included “Takes time to visit the hospital” and “Risk of infectious diseases.” Word clouds illustrating the advantages of both OGC and IPGC are shown in Figure 2. The OGC word cloud predominantly featured terms focusing on practical advantages such as “time,” “travel,” “convenient,” “infection,” and “cost.” In contrast, the IPGC word cloud emphasized terms related to interpersonal aspects, including “feel,” “communication,” “emotions,” “atmosphere,” “reassurance,” “anxieties,” and “conversation.” Taken together, OGC was valued for its practical advantages in terms of access and convenience, whereas IPGC was preferred for the perceived quality of interpersonal communication and emotional connection.

**Table 2.** Categorical Classification of Advantages and Disadvantages in OGC and IPGC.

	OGC	IPGC
Advantages	Easy access even from remote areas (6)	Emotions, facial expressions, and atmosphere are easily conveyed (5)
	Saves time (5)	Smooth communication (4)
	Good communication possible (5)	No communication setup or technical issues (3)
	No need to travel (4)	Provides a sense of security (3)
	Easy to receive genetic counseling (3)	Easy to ask questions (1)
	No risk of infection (2)	Physical examination possible (1)
	Saves money (2)	No inconvenience with in person genetic counseling (1)
	Easier for family to join (1)	Blood tests can be performed on the same day (1)
		Easier to speak honestly (1)
		Can view materials while listening to explanations (1)
		
Disadvantages	Difficulty ensuring privacy protection (2)	Takes time to visit the hospital (2)
	Problems accessing online systems (1)	Risk of infectious diseases (1)
	Difficulty in communication (1)	
	Unable to perform physical examinations (1)	
	Blood tests not possible on the same day (1)	
	Some topics may not be suitable (1)	
	Lack of awareness among health care providers (1)	
	Materials hard to see on device screens (1)	

Numbers in parentheses indicate the number of respondents. IPGC, in-person genetic counseling; OGC, online genetic counseling.

## Discussion

In this study, all participants in the OGC group expressed satisfaction with their genetic counseling sessions by selecting either “Strongly agree” or “Agree,” indicating high satisfaction levels. The participants also identified numerous benefits of OGC, supporting our hypothesis that this modality could contribute to reducing disparities in access to genetic medicine services in Japan. However, participants in the IPGC group showed significantly higher scores in overall session satisfaction, and the free-text responses revealed several challenges associated with OGC.

### Satisfaction with OGC

Our findings suggest that overall satisfaction with OGC was high. It is noteworthy that all respondents reported satisfaction with their OGC sessions. Previous studies have shown that OGC yields levels of patient satisfaction similar to those of IPGC or higher ^(7), (11), (13), (14), (15), (16)^, and our results support these findings. However, in this study, the IPGC group showed significantly higher scores in several aspects: the counselors’ self-introductions and role explanations, appropriate responses to questions, and overall session satisfaction. On the basis of the advantages of IPGC identified in the free-text responses and key terms in the word cloud analysis, these differences may reflect the enhanced quality of communication in face-to-face interactions. Indeed, previous studies have indicated that IPGC enables richer communication through nonverbal cues ^(9), (10)^ and facilitates trust-building ^(23)^. Alternatively, given the non-randomized nature of the study, it is possible that individuals with higher expectations for genetic counseling may have been more likely to select OGC, potentially yielding lower satisfaction scores in the OGC group. In contrast, for instance, individuals opting for OGC owing to significant travel burdens might have different baseline expectations regarding the counseling experience from those choosing IPGC, which could in turn affect reported satisfaction levels ^(24)^. The observed differences could be influenced by unmeasured factors related to participant selection; thus, further research with controlled designs would be needed to explore these potential influences.

### Situations that enhance OGC benefits

Our study revealed that OGC provides significant advantages for patients accessing genetic counseling services from remote locations. These benefits include not only reduced travel time but also lower travel costs and easier participation of family members from different locations. In addition, the prominence of terms such as “time,” “travel,” and “convenient” in the word cloud analysis suggests that participants in OGC highly value its potential convenience and practical benefits. These findings align with previous studies from Western countries indicating that OGC effectively reduces disparities in access to genetic specialists by addressing temporal and financial barriers ^(9), (10), (11), (12), (23)^.

Notably, in this study, approximately 35% of participants in OGC had hereditary retinal diseases. Previous research has shown that more than 90% of patients with hereditary retinal diseases who used remote counseling were satisfied with their sessions, with more than half reporting improved access to care ^(25)^. In this context, improved access to care refers not only to the reduction of physical distance but also to the alleviation of the burden associated with traveling to specialized genetic counseling facilities, and reductions in time and financial costs. These improvements, in turn, increase opportunities for patients to consult with specialists. In other words, they enable patients to access the necessary information and professionals when needed. These findings suggest that OGC can be particularly beneficial for patients with conditions that make travel difficult. In addition to travel challenges, patients with visual impairment may further benefit from OGC using their own familiar devices with personalized settings, such as screen brightness, magnification, or color inversion, which can help reduce visual strain often caused by unfamiliar clinical equipment.

### Challenges in implementing OGC

Through this study, several challenges associated with OGC implementation were identified: “difficulty ensuring privacy protection,” “problems accessing online systems,” “difficulty in communication,” and “materials hard to see on device screens.” In Western countries where OGC is widely practiced, challenges such as difficulties in nonverbal communication and internet connectivity issues have been reported ^(9), (10)^. In this study, in addition to these previously identified challenges, privacy concerns and screen visibility issues were also noted, highlighting the need for further investment in secure, user-friendly, and high-quality ICT infrastructure. Notably, not all participants perceived these challenges as significant, suggesting that individual experiences may vary depending on factors such as internet infrastructure, devices used, digital literacy, and personality. Although no significant differences were observed, it is also worth noting that the OGC group included a relatively higher proportion of younger participants, who are likely to have higher digital literacy.

Furthermore, cultural and institutional factors need to be carefully considered when implementing OGC in Japan. Japanese individuals often exhibit heightened sensitivity regarding familial matters and show a distinct hesitancy toward genetics-related topics ^(19)^. These cultural characteristics may pose additional barriers to the adoption of OGC compared with other countries, particularly owing to privacy concerns inherent in telemedicine. Furthermore, as of 2025, Japan’s national health insurance system provides coverage for OGC only under very limited conditions, making its routine implementation challenging. In this study, none of the OGC sessions were covered by national health insurance, whereas some IPGC sessions, associated with specific genetic tests or conditions such as hereditary breast and ovarian cancer syndrome, were reimbursed. This highlights a significant disparity in payment structure that could hinder the broader adoption of OGC.

Moreover, health care professionals, as service providers rather than recipients, have faced several operational challenges in implementing OGC, including time-intensive preparation of materials, network setup, and the establishment of payment systems. Although OGC provides clear benefits for patients, it imposes significant workload and logistical burdens on genetic counseling staff and other health care personnel. Without adequate institutional support, resources, and potentially financial or structural incentives to offset these burdens, widespread and sustainable implementation may be compromised. Accordingly, health care policy reforms, including the expansion of insurance coverage, the development of regulatory frameworks and clinical guidelines, and the establishment of standardized best practices for OGC, will be essential to promote its broader adoption within the Japanese health care system.

It is important to acknowledge that although this study provides valuable insights into client satisfaction with OGC in Japan, a comprehensive assessment of OGC as a viable alternative or complement to IPGC requires a multifaceted approach. This study did not assess health care providers’ views on the operational challenges previously discussed. Future research in the Japanese context should also incorporate the perspectives of health care providers on the feasibility, benefits, and challenges of delivering OGC. Furthermore, broader considerations related to health economics, integration into existing health care systems, data security frameworks beyond individual user concerns, and evolving ethical guidelines for telemedicine in genetics are crucial. Our study contributes by establishing a foundational understanding of patient experiences and preferences in Japan, a critical first step toward establishing a comprehensive framework appropriately tailored to the Japanese health care context for delivering OGC.

On the basis of our findings, several practical improvements could be considered for our institution and similar settings implementing or expanding OGC services in Japan. First, to address nuances in communication, standardized introductory protocols for OGC―clearly explaining the counselor’s role and session flow, potentially supplemented with pre-session visual aids―could enhance patient preparedness and comfort, thereby improving scores related to counselor introduction and responsiveness. Second, to alleviate privacy concerns, providing patients with clear and accessible information about the security features of the OGC platform, along with guidance on creating a private setting for their session, may be helpful. Third, investing in user-friendly platforms and offering optional brief training or troubleshooting guides for patients less familiar with technology could reduce technical barriers. Finally, systematically collecting feedback from both patients and OGC providers would allow iterative refinement of the service, addressing emerging challenges such as material visibility, communication flow, and administrative burden, thereby promoting a more effective and sustainable OGC program.

### Study limitations

This study has several limitations. First, the small number of participants, particularly in the OGC group, and the single-center design limit the generalizability of our findings to other health care settings or broader patient populations in Japan. Second, OGC was provided on the basis of patient preference rather than random assignment, introducing potential selection bias. Participants who chose OGC may have had pre-existing characteristics (e.g., greater comfort with technology or stronger motivation to avoid travel) that differed from those who chose IPGC or the general population ^(24)^. As a result, their high ratings of convenience and overall satisfaction may not be fully generalizable. However, the benefit of OGC for individuals with long travel distances likely represents a genuine advantage for this subgroup. Moreover, given some IPGC sessions were covered by insurance reimbursement, it is possible that the out-of-pocket expenses for participants differed between OGC and IPGC, which could have influenced satisfaction ratings. Third, the data collection period (July 2020 to January 2025) coincided with the COVID-19 pandemic, during which telemedicine rapidly expanded from necessity ^(16), (18)^. This situation may have influenced participants’ choices and satisfaction―for example, OGC may have been perceived more favorably owing to safety concerns or limited access to in-person care. These pandemic-specific factors should be considered when interpreting findings in the post-pandemic era. Consequently, larger-scale, multi-center studies are necessary to provide further insights into OGC, particularly in the post-pandemic context.

### Conclusion

OGC shows considerable potential to address unmet needs in genetic medicine in Japan by improving accessibility, reducing travel burden, and enhancing patient convenience. Although participants in OGC reported high satisfaction, IPGC was preferred for its richer communication. Privacy concerns, technical challenges, and limited insurance coverage remain substantial barriers to its widespread implementation. Future efforts should prioritize refining OGC frameworks, advancing ICT infrastructure, establishing comprehensive guidelines and best practices, and implementing necessary policy reforms. Further research and clinical validation are essential for the successful integration of OGC into the Japanese health care system.

## Article Information

### Acknowledgments

We appreciate the participation and cooperation of all individuals involved in the study.

### Author Contributions

Kazuki Yamazawa conceived the study. Haruka Murakami and Satomi Inoue collected the questionnaire forms. Haruka Murakami compiled the data and conducted the statistical analysis. Haruka Murakami and Satomi Inoue conducted the conventional content analysis. Kaoru Fujinami, Tatsuo Matsunaga, and Kazuki Yamazawa provided genetic counseling sessions and supervised the study. Haruka Murakami and Kazuki Yamazawa drafted the manuscript. All authors reviewed and approved the final version of the manuscript.

### Conflicts of Interest

None

### IRB Approval

This study was approved by the institutional review board of the NHO Tokyo Medical Center (Approval Number: R22-115).

## Supplement

Supplementary Material Supplementary information
Supplementary Document 1. Original Questionnaire for OGC
Supplementary Document 2. Original Questionnaire for IPGC
Supplementary Table 1. Communication Devices and Connection Quality (OGC Group Only).
