# Supplementary material for: Online Genetic Counseling as a Solution for Unmet Needs in Genetic Medicine: The First Survey in Japan
Source: JMA J. 2025 Nov 21;9(1):160–70. doi: 10.31662/jmaj.2025-0157 (PMC12889013; doi:10.31662/jmaj.2025-0157)
Supplement: Supplementary Material — Supplementary information Supplementary Document 1. Original Questionnaire for OGC Supplementary Document 2. Original Questionnaire for IPGC Supplementary Table 1. Communication Devices and Connection Quality (OGC Group Only). [file 2433-3298-9-1-0160-s001.pdf]

Supplementary document 1

Questionnaire for online genetic counseling

· The results of this survey will be used solely for the purpose of improving the quality of online genetic counseling.

· This survey is not intended to identify individuals.

· After completing the questionnaire, please place it in the enclosed return envelope and mail it anonymously.

Date of Completion: Year______ Month______ Day______

Please circle the most applicable options or fill in the blanks as appropriate.

Gender: Male / Female

Age: 20s / 30s / 40s / 50s / 60s / 70 or older

Consultation Topic: Hereditary hearing loss / Hereditary retinal disease / Hereditary cancer
Congenital pediatric disorders / Other (______________________________)

Relationship to the Individual with Symptoms:

Self / Parent / Child / Sibling / Grandparent / Cousin / Other (_________________)

Number of Genetic Counseling Sessions: First / Second / Third / Fourth or more

Travel Time to the Hospital: Under 30 minutes / 30-60 minutes / 1-2 hours / Over 2 hours

(1) Please circle the number that best applies to you.

	Strongly Agree	Agree	Neutral	Disagree	Strongly Disagree
**Regarding Genetic Counseling**					
Q1. I understood my genetic risks even before the counseling session.	1	2	3	4	5
Q2. The counselor introduced themselves and explained their role before starting the session.	1	2	3	4	5
Q3. The counselor listened to and respected the information I provided.	1	2	3	4	5
Q4. The counselor provided the information I needed.	1	2	3	4	5
Q5. The counselor appropriately answered my questions.	1	2	3	4	5
Q6. Personal information about me and my family was kept confidential.	1	2	3	4	5
Q7. I am satisfied with the advice I received today.	1	2	3	4	5
Q8. I would recommend this genetic counseling session to other family members.	1	2	3	4	5
Q9. If possible, I would prefer to have in-person genetic counseling for the next session.	1	2	3	4	5
Q10. I believe that satisfaction levels for online and in person genetic counseling are the same.	1	2	3	4	5
Q11. Overall, I am satisfied with today's genetic counseling session.	1	2	3	4	5

	Strongly Agree	Agree	Neutral	Disagree	Strongly Disagree
**Regarding Communication Devices and Connection Quality**					
Q1. The communication device was delivered to my home without any issues.	1	2	3	4	5
Q2. The communication device was easy to use.	1	2	3	4	5
Q3. During the online genetic counseling session, the counselor's voice was clear.	1	2	3	4	5
Q4. The screen was stable during the online genetic counseling session.	1	2	3	4	5
Q5. I felt comfortable asking questions during the online genetic counseling session.	1	2	3	4	5
Q6. I was concerned about the risk of personal information being leaked during the session.	1	2	3	4	5
Q7. Returning the communication device was easy.	1	2	3	4	5

(2) Compared to in-person genetic counseling, if you noticed any advantages or disadvantages of online genetic counseling, please share them freely.

(3) Please feel free to share your thoughts and requests regarding today's genetic counseling session.

Thank you very much for your cooperation.

Supplementary document 2

Questionnaire for in-person genetic counseling

- The results of this survey will be used solely for the purpose of improving the quality of online genetic counseling.
- This survey is not intended to identify individuals.
- After completing the questionnaire, please place it in the enclosed return envelope and mail it anonymously.

Date of Completion: Year______ Month______ Day______

Please circle the most applicable options or fill in the blanks as appropriate.

Gender: Male / Female

Age: 20s / 30s / 40s / 50s / 60s / 70 or older

Consultation Topic: Hereditary hearing loss / Hereditary retinal disease / Hereditary cancer
Congenital pediatric disorders / Other (______________________________)

Relationship to the Individual with Symptoms:

Self / Parent / Child / Sibling / Grandparent / Cousin / Other (_________________)

Number of Genetic Counseling Sessions: First / Second / Third / Fourth or more

Travel Time to the Hospital: Under 30 minutes / 30-60 minutes / 1-2 hours / Over 2 hours

(1) Please circle the number that best applies to you.

	Strongly Agree	Agree	Neutral	Disagree	Strongly Disagree
**Regarding Genetic Counseling**					
Q1. I understood my genetic risks even before the counseling session.	1	2	3	4	5
Q2. The counselor introduced themselves and explained their role before starting the session.	1	2	3	4	5
Q3. The counselor listened to and respected the information I provided.	1	2	3	4	5
Q4. The counselor provided the information I needed.	1	2	3	4	5
Q5. The counselor appropriately answered my questions.	1	2	3	4	5
Q6. Personal information about me and my family was kept confidential.	1	2	3	4	5
Q7. I am satisfied with the advice I received today.	1	2	3	4	5
Q8. I would recommend this genetic counseling session to other family members.	1	2	3	4	5
Q9. If possible, I would prefer to have online genetic counseling for the next session.	1	2	3	4	5
Q10. I believe that satisfaction levels for online and in person genetic counseling are the same.	1	2	3	4	5
Q11. Overall, I am satisfied with today's genetic counseling session.	1	2	3	4	5

(2) Compared to online genetic counseling, if you noticed any advantages or disadvantages of in-person genetic counseling, please share them freely.

(3) Please feel free to share your thoughts and requests regarding today's genetic counseling session.

Thank you very much for your cooperation.

Supplementary table 1. Communication devices and connection quality (OGC group only)

	n (%)
**Q1. The communication device was delivered to my home without any issues.**	
Strongly agree	2 (13.3)
Agree	1 (6.7)
Neutral	2 (13.3)
Disagree	1 (6.7)
Strongly disagree	2 (13.3)
Not specified	7 (46.7)
**Q2. The communication device was easy to use.**	
Strongly agree	4 (26.7)
Agree	2 (13.3)
Neutral	3 (20)
Disagree	1 (6.7)
Strongly disagree	0 (0)
Not specified	5 (33.3)
**Q3. During the online genetic counseling session, the counselor's voice was clear.**	
Strongly agree	4 (26.7)
Agree	9 (60)
Neutral	1 (6.7)
Disagree	0 (0)
Strongly disagree	0 (0)
Not specified	1 (6.7)
**Q4. The screen was stable during the online genetic counseling session.**	
Strongly agree	5 (33.3)
Agree	8 (53.3)
Neutral	0 (0)
Disagree	1 (6.7)
Strongly disagree	0 (0)
Not specified	1 (6.7)
**Q5. I felt comfortable asking questions during the online genetic counseling session.**	
Strongly agree	8 (53.3)
Agree	5 (33.3)
Neutral	1 (6.7)
Disagree	0 (0)
Strongly disagree	0 (0)
Not specified	1 (6.7)
**Q6. I was concerned about the risk of personal information being leaked during the session.**	
Strongly agree	1 (6.7)
Agree	0 (0)
Neutral	2 (13.3)
Disagree	5 (33.3)
Strongly disagree	6 (40)
Not specified	1 (6.7)
**Q7. Returning the communication device was easy.**	
Strongly agree	0 (0)
Agree	1 (6.7)
Neutral	4 (26.7)
Disagree	0 (0)
Strongly disagree	1 (6.7)
Not specified	9 (60)

OGC, online genetic counseling.
